# Preconditioning of bone marrow-derived mesenchymal stem cells highly strengthens their potential to promote IL-6-dependent M2b polarization

**DOI:** 10.1186/s13287-018-1039-2

**Published:** 2018-10-25

**Authors:** Denise Philipp, Laura Suhr, Thorsten Wahlers, Yeong-Hoon Choi, Adnana Paunel-Görgülü

**Affiliations:** Department of Cardiothoracic Surgery, Heart Center of the University of Cologne, Cologne, Germany

**Keywords:** Mesenchymal stem cells, Macrophage polarization, IL-6, Preconditioning, Immunosuppression

## Abstract

**Background:**

During the last decade, mesenchymal stem cells (MSCs) have gained much attention in the field of regenerative medicine due to their capacity to differentiate into different cell types and to promote immunosuppressive effects. However, the underlying mechanism of MSC-mediated immunoregulation is not fully understood so far. Macrophages are distinguished in classical activated, pro-inflammatory M1 and alternatively activated M2 cells, which possess different functions and transcriptional profiles with respect to inflammatory responses. As polarization is not fixed, macrophage functional plasticity might be modulated by the microenvironment allowing them to rapidly react to danger signals and maintaining tissue homeostasis.

**Methods:**

Murine MSCs were preconditioned with IL-1ß and IFN-ɣ to enhance their immunosuppressive capacity regarding macrophage polarization under M1- and M2a-polarizing conditions. Macrophage polarization was analyzed by real-time PCR, flow cytometry, and cytokine detection in culture supernatants. The role of MSC-derived nitric oxide (NO), prostaglandin E2 (PGE2), and IL-6 in this process has been evaluated using siRNA transfection and IL-6 receptor-deficient macrophages, respectively.

**Results:**

Preconditioned, but not unprimed, MSCs secreted high levels of NO, IL-6, and PGE2. Co-culture with macrophages (M0) in the presence of M1 inducers (LPS + IFN-ɣ) led to significant reduction of CD86 and iNOS protein in macrophages and diminished TNF-α secretion. Additionally, CD86 and iNOS protein expression as well as NO and IL-10 secretion were markedly increased under M2a-polarizing culture conditions (IL-4). MSC-dependent macrophage polarization did not depend on direct cell-cell contact. Co-culturing in the presence of LPS and IFN-ɣ resulted in the upregulation of M2a, M2b, and M2c marker genes, whereas in the presence of IL-4 only M2b markers were significantly increased. In turn, IL-10-producing regulatory M2b cells significantly inhibited IFN-ɣ expression in CD4^+^ T lymphocytes. Finally, we show that MSC-mediated macrophage polarization strongly depends on IL-6, whereas a minor role for NO and PGE2 was found.

**Conclusions:**

Preconditioning of MSCs highly strengthens their capacity to regulate macrophage features and to promote immunosuppression. Repression of M1 polarization during inflammation and M2b polarization under anti-inflammatory conditions strongly depend on functional IL-6 signaling in macrophages. The potential benefit of preconditioned MSCs and IL-6 should be considered for future clinical treatment.

**Electronic supplementary material:**

The online version of this article (10.1186/s13287-018-1039-2) contains supplementary material, which is available to authorized users.

## Background

Bone marrow-derived mesenchymal cells (MSCs) are pluripotent adult stromal cells able to differentiate into different cell types such as osteoblasts, chondrocytes, adipocytes, myocytes, and neurons [1–3]. MSCs preferentially recruit to sites of tissue damage, and several studies have demonstrated that these cells are capable of producing a wide range of growth factors such as transforming growth factor-β (TGF-β), hepatocyte growth factor (HGF), epidermal growth factor (EGF), fibroblast growth factor (FGF), vascular endothelial growth factor (VEGF), platelet-derived growth factor (PDGF), insulin-like growth factor 1 (IGF-1), stromal cell-derived factor 1 (SDF-1), and angiopoietin-1 [4] which support tissue regeneration and repair. Due to their ability of self-renewal and to differentiate into multiple tissues, MSCs have attracted most scientific attention as potential therapeutic tools for cell-based therapy. Recently, it became evident that MSCs exert potent immunomodulatory capacities. In general, MSC-mediated immunosuppression requires preliminary activation of MSCs by immune cells through the secretion of interferon gamma (IFN-ɣ) as part of the inflammatory milieu [5]. The important role of the IFN-ɣ-triggered, essential activation step is highlighted by the finding that MSCs from IFN-ɣ^−/−^ mice do not have immunosuppressive activity [6, 7]. The mechanisms underlying these immunosuppressive effects mediated by MSCs are actually not fully understood, but they appear to be mediated by soluble factors, produced constitutively or in response to paracrine signals, by MSCs. These soluble mediators include, e.g., transforming growth factor beta 1 (TGF-β1), prostaglandin E2 (PGE2), indoleamine 2,3-dioxygenase (IDO), nitric oxide (NO), heme oxygenase, interleukin (IL)-6, and IL-10 [8–10]. The effects of MSCs on immune cells are multifarious. MSCs have been demonstrated to inhibit monocyte differentiation into dendritic cells (DCs), to suppress the maturation of DCs as well as the production of IFN-ɣ by NK cells and to exert strong immunosuppressive effects on T [9] and B cells [11], among others. Several studies reported functional interaction between MSCs and macrophages as well as regulation of macrophage function by MSCs. Macrophages can acquire distinct functional phenotypes via undergoing different phenotypic polarization. Classically activated M1 macrophages show pro-inflammatory properties because they express high levels of inducible nitric oxide synthase (iNOS) activity and release large amounts of NO, tumor necrosis factor alpha (TNF-α), IL-1ß, and IL-12. Within the anti-inflammatory M2 set, several subsets characterized by unique functions are distinguished depending on the polarizing stimuli such as M2a, M2b, M2c, and M2d (only in mice) [12]. M2 macrophages are observed in healing-type circumstances promoting wound healing and tissue remodeling and generate anti-inflammatory cytokines such as IL-10 and relatively low levels of TNF-α or IL-12, respectively [13]. Through the actions of MSCs, monocytes are directed to differentiate into alternative anti-inflammatory M2 phenotype. Therefore, MSCs have the potential to suppress uncontrolled immune responses, by in situ downregulation of the inflammatory response. In this study, we have explored the immunosuppressive effects of bone marrow-derived MSCs in respect to their ability to promote a shift in macrophage phenotype in dependence of their preconditioning with pro-inflammatory cytokines. We hypothesize that pretreatment by inflammatory cytokines should be a better strategy for future application of MSCs in clinic.

## Methods

### Animals

In this study, adult 8–12-week-old C57/Bl6 and IL-6Rα-deficient [14] C57/Bl6 mice were used. Mice were maintained in the local animal facility at a 12-h light/dark cycle with free access to food and water.

### Isolation and characterization of bone marrow-derived MSCs

MSCs were isolated from femurs of 10–12-week-old C57Bl/6 mice. Bone marrow cells were obtained by flushing out femurs with PBS. Cells were cultured in a density of 1.5–2 × 10^6^/cm^2^ in MSC culture medium (PAN-Biotech) supplemented with 2.5 ng/ml human basic fibroblast growth factor FGF (FGF-b, Peprotech), 100 U/ml penicillin, and 10 μg/ml streptomycin (Sigma Aldrich) at 37 °C until a proliferative and homogenous MSC population was obtained. To prove MSC status, cells were differentiated along adipogenic, chondrogenic, and osteogenic lineages by means of differentiation medium as previously described [15].

In addition, MSC-specific surface markers were analyzed by incubation of cells with monoclonal fluorochrome-labeled antibodies against stem-cell antigen 1 (PE rat anti-mouse Sca-1, clone E13-161.7, BD), CD29 (PE hamster anti-mouse, clone HM β1-1, BD), CD44 (FITC rat anti-mouse, clone IM7, BD), and CD49e (PE rat anti-mouse, clone 5H10-27, BD) for 20 min at 4 °C in cellwash buffer (BD) supplemented with 3% FCS. Antibodies against CD11b (PE rat anti-mouse, clone M1/70, BD) and CD45 (FITC rat anti-mouse, clone 30-F11, BD) were used to exclude the expression of these molecules on our MSC population. After washing with 3% FCS in cell wash buffer (BD), cells were analyzed by flow cytometry using a MACSQuant Analyzer (Miltenyi Biotec).

For in vitro cell activation of bone marrow-derived MSCs, MSCs were treated with 30 ng/ml recombinant murine IFN-γ (Peprotech) and 3 ng/ml recombinant murine IL-1β (Peprotech) in MSC medium supplemented with 2.5 ng/ml FGF-b for 24 h. MSCs without cytokine treatment served as controls.

### Isolation, in vitro polarization, and characterization of murine macrophages

Bone marrow cells were isolated by flushing femurs of 8–12-week-old C57/Bl6 or IL-6Rα-deficient C57/Bl6 mice, respectively. Cells (1.7–2 × 10^6^) were cultured in six-well plates in RPMI medium supplemented with 20% FCS containing 20 ng/ml recombinant murine M-CSF (Peprotech), 100 U/ml penicillin, and 10 μg/ml streptomycin (Sigma Aldrich) in a humidified incubator at 37 °C. On day 3 and day 6, the medium was changed. Differentiated macrophages (M0) were obtained after 7 days of culture. Expression of the macrophage markers CD11b and F4/80 was proven by flow cytometry using PE- and FITC-labeled antibodies (PE rat anti-mouse, clone M1/70, BD; APC rat anti-mouse, clone BM8, Bio Legend). For in vitro polarization, M0 macrophages were cultured in RPMI medium with 10% FCS supplemented with 20 ng/ml recombinant murine IFN-ɣ (Peprotech) and 100 ng/ml LPS (Sigma Aldrich) to induce a M1-like phenotype or in medium supplemented with 20 ng/ml recombinant murine IL-4 (Peprotech) to induce M2a-like macrophages, respectively. In some experiments, macrophages were additionally treated with 25 ng/ml recombinant murine IL-6 (Peprotech).

After 24 h, the phenotype of macrophages was analyzed by flow cytometry (MACSQuant Analyzer, Miltenyi Biotec). The following fluorochrome-labeled antibodies were used: CD11b (PE rat anti-mouse, clone M1/70, BD), F4/80 (APC rat anti-mouse, clone BM8, Bio Legend), CD86 (FITC rat anti-mouse, clone GL1, BD), CD206 (Alexa Fluor 647 rat anti-mouse, clone MR5D3, BD), and IL6Rα (PE anti-mouse CD126 (IL-6Rα chain), clone D7715A7, Bio Legend). The macrophage phenotypes were further examined by real-time PCR and ELISA.

### Isolation of CD4^+^ T lymphocytes

CD4^+^-positive lymphocytes were isolated from spleens of C57/Bl6 wild type mice. Single-cell suspensions were obtained by using gentle MACS C tubes and gentle MACS dissociator according to the protocol provided by the company (Miltenyi Biotec). Spleen isolation and cell labeling were performed under sterile conditions. The spleens were isolated and transferred into gentle MACS C tubes containing sterile PBS supplemented with 0.5% BSA and 2 mM EDTA. Using the gentle MACS dissociator, the spleens were segregated. The cell suspension was then transferred into a 50-ml tube using a 70-μm filter and centrifuged at 1000 rpm for 2 min. After that, the cells were washed using 5 ml PBS supplemented with 0.5% BSA and 2 mM EDTA and centrifuged at 400×*g* for 10 min. Following, residual erythrocytes were removed by resuspending the cell pellet in 6 ml 0.2% NaCl for 45 s. The lysis was then stopped by adding 14 ml 1.2% NaCl. The cell suspension was filtered, centrifuged, and counted, and cells were labeled for negative depletion of CD4^+^ cells. According to the protocol provided by Miltenyi Biotec, the cells were labeled with biotin-antibody cocktail and incubated for 5 min at 4 °C. After that, anti-biotin conjugated microbeads were added and incubated for 10 min at 4 °C. Finally, the CD4^+^-positive lymphocytes were separated by negative depletion using autoMACS (Miltneyi Biotec). Purity of CD4^+^ cells was confirmed by flow cytometry using PE-conjugated CD4 antibody (clone YTS 191.1.2, ImmunoTools).

### Co-culture of bone marrow-derived MSCs and macrophages

MSCs and macrophages (M0) were suspended in RPMI medium supplemented with 10% FCS, 100 U/ml penicillin, 10 μg/ml streptomycin, and M1 or M2 activating cytokines, respectively. Cells were cultured in six-well plates at a MSC:M ratio of 1:2 (2.5 × 10^5^ MSC and 5 × 10^5^ M) for 24 h. Controls of macrophages and MSCs cultured alone were included. In parallel experiments, MSCs were preconditioned with 30 ng/ml IFN-γ and 3 ng/ml IL-1β for 24 h before culturing with macrophages. After co-culture, cells were separated using magnetic separation (autoMACS, Miltneyi Biotec) by following the manufacturer’s instructions. In brief, macrophages were labeled with a biotin-conjugated F4/80 antibody (Miltenyi Biotec) for 10 min at 4 °C and then further incubated with monoclonal anti-biotin microbeads UltraPure (Miltenyi Biotec) for 15 min at 4 °C. After washing of cells, cells were loaded onto AutoMACS columns (Miltneyi Biotec) and non-labeled cells (MSCs) were collected at the outlet port “negative” whereas labeled macrophages were eluted at the positive outlet. Cells were immediately examined by flow cytometry.

For transwell experiments, bone marrow-derived cells were seeded into six-well plates and allow to differentiate into M0 macrophages as described above. At day 7, MSCs were placed into 0.4 μm inserts (MSC:M ratio 1:2) and cells were further cultured in the presence of M1 and M2 inducers for 24 h at 37 °C. Supernatants were collected and stored at − 80 °C for further analyses. Cells were immediately analyzed by flow cytometry.

### Co-culture of macrophages with CD4^+^ T lymphocytes

Macrophages pre-cultured in transwells with preconditioned MSCs under M2a polarizing conditions were further cultured with purified CD4^+^ T cells (1 × 10^6^ cells per well). Anti-mouse CD3e (1 μg/ml, clone 145-2C11, eBioscience) and anti-mouse CD28 (1 μg/ml, clone 37.51, eBioscience) were added to the co-culture system, and cells were incubated for 24 h at 37 °C. CD4^+^, non-adherent cells were harvest from the co-culture supernatants by gentle pipetting and lysed for RNA extraction.

### siRNA transfection

To knockdown *iNOS* and *COX-2* expression, 5 × 10^4^ MSCs were seeded in six-well plates 2 days before transfection. Transfection has been performed using OptiMEM medium (Gibco) and Lipofectamine RNAiMAX Reagent (Invitrogen) according to manufacturer’s instructions. Cells were transfected with 5.5 nM Silencer Select iNOS siRNA, Silencer Select COX-2 siRNA, and Silencer Select negative control siRNA (Ambion), respectively, and incubated for 24 h at 37 °C and 5% CO_2._ MSCs were further activated by treatment with 30 ng/ml recombinant murine IFN-γ (Peprotech) and 3 ng/ml recombinant murine IL-1β (Peprotech) for additional 24 h. Transfected, preconditioned cells were used for co-culture experiments. Transfection efficiency was proven by real-time PCR, ELISA, and Griess Assay.

### Real-time PCR

Total RNA was extracted using RNeasy Mini Kit (Qiagen) according to the manufacturer’s instructions. Contaminating DNA was removed by DNA-*free* Kit DNA Removal Kit (Ambion). RNA was reverse transcribed using High-Capacity cDNA Reverse Transcription Kit (Applied Biosystems). For real-time PCR, gene-specific primers for *iNOS* [16], *Arg I* [17], *COX-2* [18], *TNF-α* [19], *Fizz1/RELMα*, *Ym-1* [20], *SPHK1*, *LIGHT* [21], *MertK* [22], *IFN-ɣ*, *IL-2*, *IL-4* [23], *IL-10* [24], and *GATA-3* [25] were used. To detect *IL-6* and *18S* RNA expression, the following primers were used: IL-6 forward 5′-CCACTTCACAAGTCGGAGGCTTA-3′; IL-6 reverse 5′-GCAAGTGCATCATCGTTGTTCATAC-3′; 18S RNA forward 5′-CGGCTACCACATCCAAGGAA-3′, 18S RNA reverse 5′-GCTGGAATTACCGCGGCT-3′.

All samples were run in triplicates. Relative gene expression levels were determined using Power SYBR Green PCR Master Mix (Applied Biosystems) according to the manufacturer’s recommended protocol with following thermal cycling conditions: 10 min 95 °C, 40 cycles of 15 s 95 °C and 60 s 60 °C, and 4 °C hold (StepOnePlus Real-Time PCR System, Applied Biosystems). Expression of target genes was normalized to the endogenous control *18S* RNA gene. Fold expression was calculated using the 2^−ΔΔCT^ method [26].

### Immunoblot analysis

Cells were lysed in RIPA buffer supplemented with protease inhibitor cocktail and phosphatase inhibitor cocktail (Cell Signaling Technology). Cell lysates were loaded on 10% polyacrylamide gel and blotted onto nitrocellulose membrane. Membranes were further blocked with blocking buffer and incubated with pSTAT3(Tyr705) antibody (clone D3A7, Cell Signaling Technology) according to the manufacturer’s instructions. Afterwards, membranes were incubated with a HRP-conjugated goat anti-rabbit secondary antibody (Dako) and developed with UptiLight HRP Blot Chemiluminescent ECL Substrate (Uptima). Blots were further stripped and reincubated with anti-STAT3 antibody (clone 9D8, Abcam) and HRP-conjugated goat anti-mouse secondary antibody (Dako).

### Analysis of cytokine production by ELISA

For cytokine quantification in culture supernatants, the following ELISA kits have been used: Human/mouse TGF beta 1 2nd Generation ELISA Ready-SET-Go (eBioscience), Mouse IL-6 DuoSet ELISA (R&D Systems), Mouse IL-10 DuoSet ELISA (R&D Systems), Mouse TNF alpha ELISA Ready-SET-Go (eBioscience), and Prostaglandin E_2_ ELISA Kit-Monoclonal (Cayman Chemical) following the manufacturers’ instructions.

### Detection of nitric oxide (NO)

NO production was quantified by measuring nitrite (NO_2_) in culture supernatants, using a modified Griess reagent (Sigma Aldrich). In brief, 80 μl supernatant was mixed with 80 μl Griess reagent and incubated at room temperature for 15 min. Absorbance at 540 nm was measured using a microplate reader (FLUOstar Omega, BMG Labtech) and nitrite concentrations were estimated using a standard nitrite curve (range 0 μM–100 μM).

### Statistical analyses

Data were analyzed with GraphPad Prism 5 software. Experimental data are presented as means with standard error of the mean (SEM). Unpaired data of two groups were analyzed using unpaired *t* test. One-sample *t* test was used when samples were compared with reference control sample (set as 1). Normally distributed unpaired data of multiple groups were analyzed using one-way ANOVA with Newman Keuls post hoc test. *p* value less than 0.05 was considered as statistically significant.

## Results

### Characterization of bone marrow-derived MSCs and polarized macrophages

**Fig. 1:**
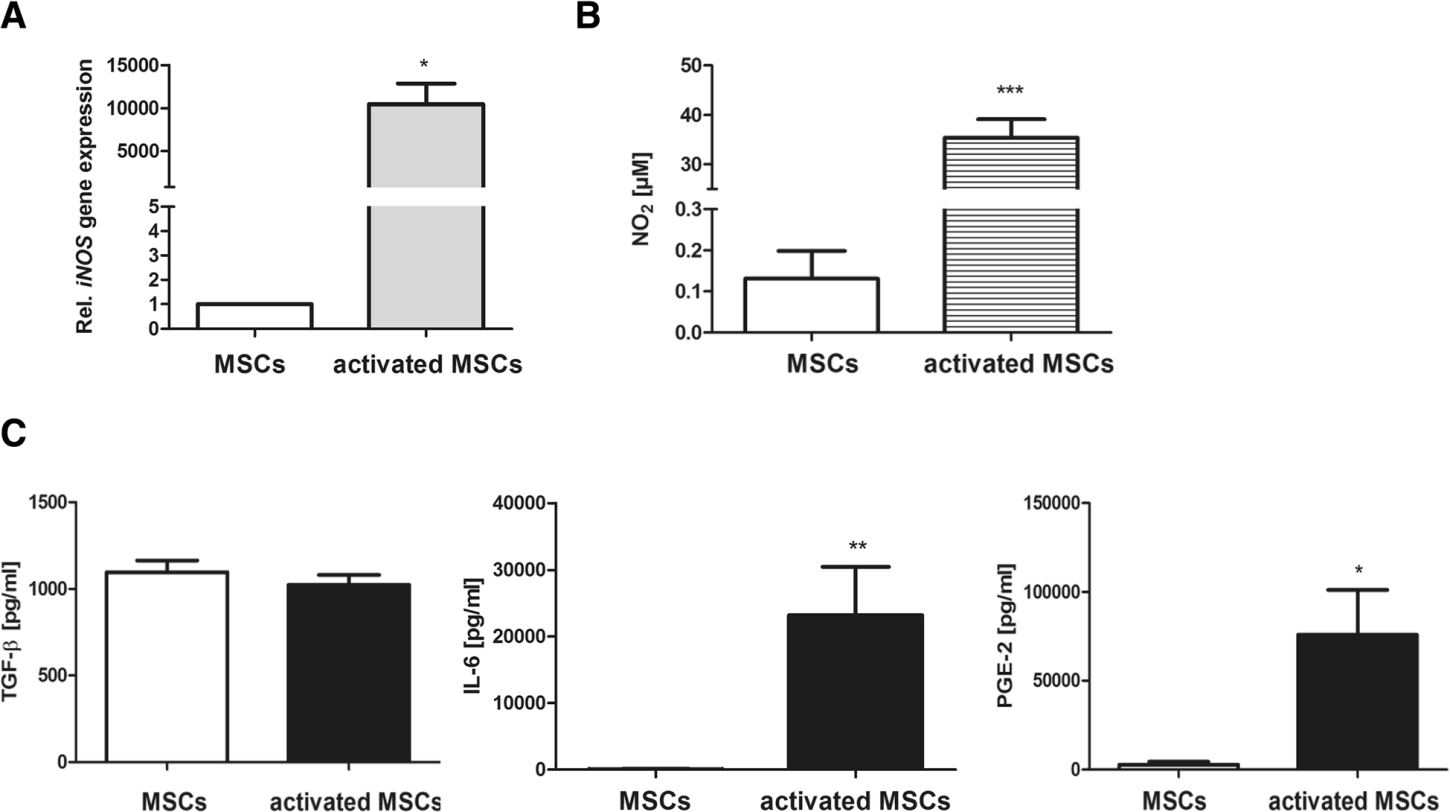
Characterization of preconditioned MSCs. MSCs were cultured in the presence of 30 ng/ml IFN-ɣ and 3 ng/ml IL-1ß for 24 h. **a** *iNOS* gene expression was analyzed by real-time PCR. *n* = 3, *p < 0.05. **b** Nitrite (NO_2_) levels in culture supernatants were quantified by Griess assay. *n* = 9, ***p < 0.001. **c** TGF-ß (*n* = 4), IL-6 (*n* = 6) and PGE2 (*n* = 5) levels in culture supernatants were quantified by ELISA. *p < 0.05; ***p < 0.001. IL interleukin, MSCs mesenchymal stem cells, iNOS inducible nitric oxide synthase, PGE2 prostaglandin E2, TGF-ß tumor growth factor beta

MSCs isolated from the bone marrow of C57/Bl6 wild type mice showed positive expression for CD29 (97.63 ± 1.3%), CD44 (65.55 ± 7.4%), CD49e (66.35 ± 6.4%), and Sca-1 (89.3 ± 4.5) and were negative for CD11b and CD45. Further on, differentiation of bone marrow-derived MSCs into adipocytes, chondrocytes, and osteoblasts was evidenced (Additional file 1). MSCs at passage ≤ p12 were used in this study. To find out if preconditioning of MSCs might alter their anti-inflammatory and immunomodulatory capacities, MSCs were initially stimulated with IL-1ß and IFN-ɣ for 24 h. As displayed in Fig. 1, cytokine stimulation strongly increased *iNOS* gene expression in MSCs (Fig. 1a), accompanied by increased amounts of secreted NO (Fig. 1b) in the culture supernatant. However, the secretion of TGF-ß, which was previously demonstrated to be a major mediator of MSC-mediated immunosuppression [27], was not influenced by the cytokine treatment. Hence, significant upregulation of IL-6 and PGE2 production by preconditioned MSCs could be observed (Fig. 1c)

### Preconditioned MSCs efficiently suppress the polarization toward the M1 phenotype

Monocyte-derived differentiated M0 macrophages were characterized in respect to the expression of F4/80 and CD11b (Additional file 2: Figure S2a). To generate different macrophage phenotypes, M0 macrophages were treated with cytokines as described under “Methods”. M1-like macrophages showed strong increase in *iNOS* expression as well as TNF-α and IL-6 secretion compared to M2a-like cells. In turn, upregulation of *Arg I* expression was detected in M2a macrophages (Additional file 2: Figure S2b, c, d).

**Fig. 2:**
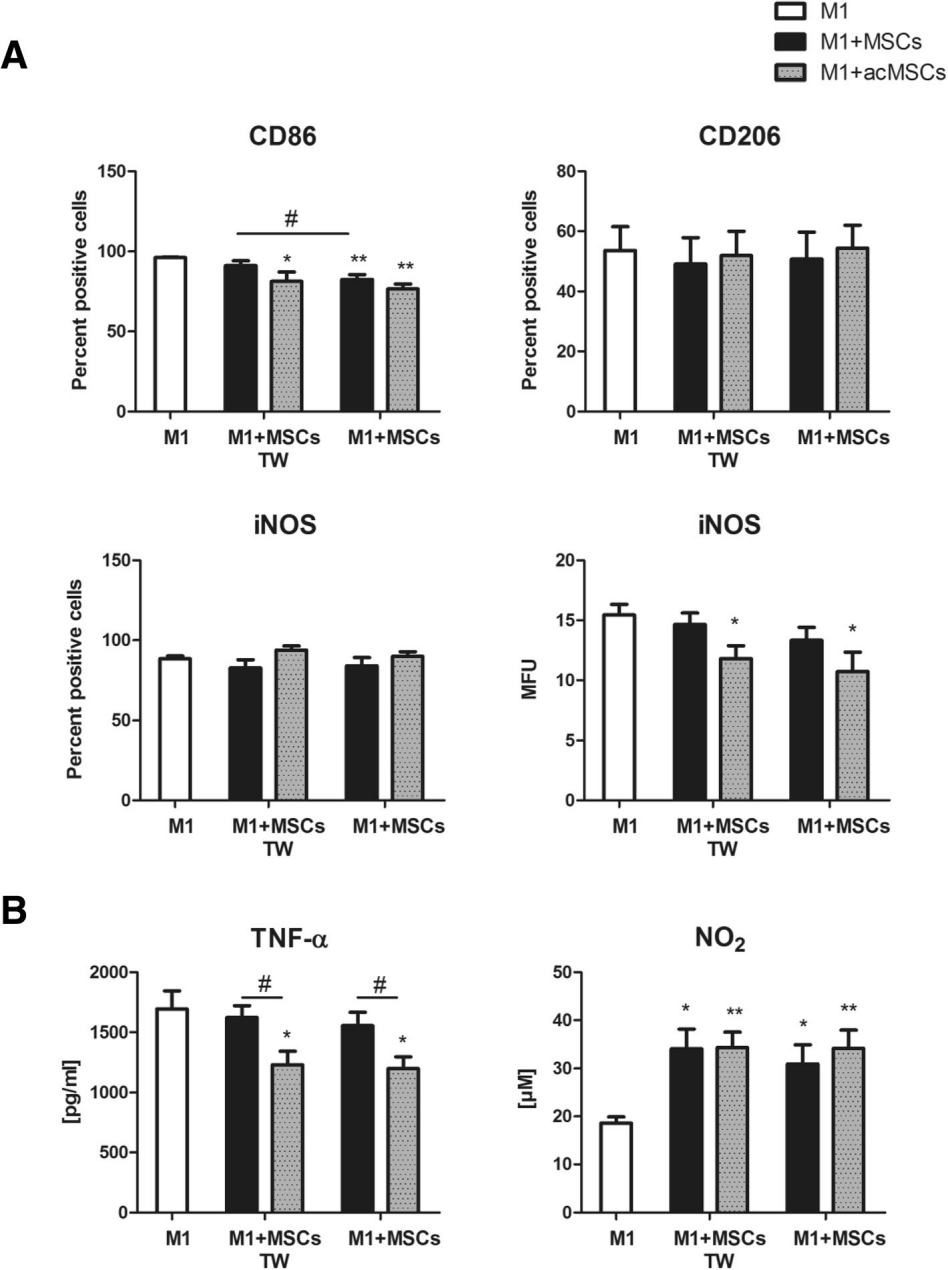
Preconditioned MSCs impair polarization toward the M1 phenotype. Unstimulated or preconditioned MSCs were co-cultured with macrophages (ratio 1:2) in the presence of M1-polarizing cytokines (20 ng/ml IFN-ɣ + 100 ng/ml LPS) for 24 h. Then, cells were separated using autoMACS. In parallel experiments, MSCs were cultured in transwells (TW) to avoid direct contact to co-cultured macrophages. **a** The expression of the surface receptors CD86 and CD206 as well as intracellular iNOS expression in macrophages was quantified by flow cytometry. Percentage of positive cells is depicted. For iNOS expression, the mean fluorescence units (MFU) are additionally displayed. *n* = 7, *p < 0.05, **p < 0.01 vs. macrophages cultured alone (M1); #*p* < 0.05. **b** TNF-α levels in culture supernatants were quantified by ELISA and NO_2_ levels were quantified by Griess assay. Results are representative for seven independent experiments. *p < 0.05, **p < 0.01 vs. macrophages cultured alone (M1); #*p* < 0.05. M macrophage, MSCs mesenchymal stem cells, iNOS inducible nitric oxide synthase, NO_2_ nitrite, TNF-α tumor necrosis factor alpha

We next examined whether preconditioning of MSCs might improve their ability to impair M1 polarization. To this end, co-culture experiments under M1-polarizing conditions were performed (M:MSC ratio 2:1). Transwell experiments were conducted in parallel to avoid direct cross-talk between MSCs and macrophages. Percentage of cells expressing co-stimulatory CD86 was significantly downregulated in the presence of MSCs, whereas the expression of the M2a marker CD206 was not influenced in co-cultures (Fig. 2a). Although the number of iNOS-positive cells did not change in the presence of preconditioned MSCs, a significant diminution of intracellular protein levels could be detected. More importantly, co-cultured preconditioned MSCs strongly suppressed TNF-α secretion indicating diminished M1 polarization (Fig. 2b). Because NO secretion is significantly increased in activated MSCs, no reduction in NO_2_ levels in culture supernatants was found (Fig. 2b), despite reduced iNOS expression in macrophages. Suppressive effects on M1 differentiation mediated by MSCs exclusively depended on soluble factors as cell separation by transwell membranes did not alter macrophage characteristics.

### Preconditioned MSCs increase CD86, iNOS, and IL-10 expression in macrophages under M2a-polarizing conditions

**Fig. 3:**
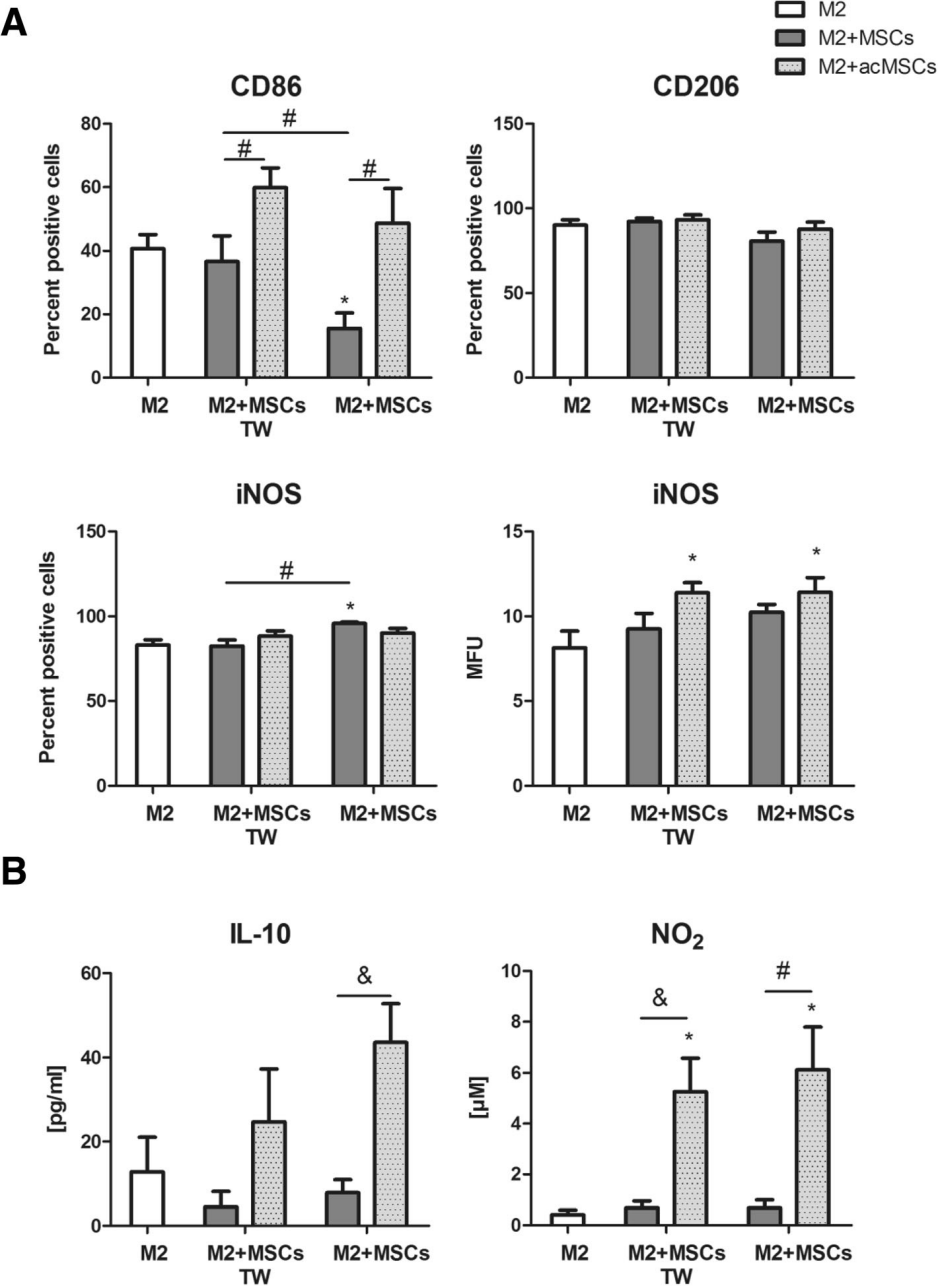
Preconditioned MSCs drive M2 polarization in the presence of IL-4. Unstimulated or preconditioned MSCs were co-cultured with macrophages (ratio 1:2) in the presence of 20 ng/ml IL-4 for 24 h. After cell separation by autoMACS, macrophages were used for flow cytometric analyses. In some experiments, MSCs were placed in transwell chambers (TW). **a** The expression of the surface markers CD86 and CD206 as well as intracellular iNOS expression in macrophages was quantified by flow cytometry. Percentage of positive cells is depicted. For iNOS expression, the mean fluorescence intensity is additionally displayed. *n* = 7, *p < 0.05 vs. macrophages cultured alone (M2); #*p* < 0.05. **b** Levels of IL-10 in cell culture supernatants were measured by ELISA. NO_2_ concentrations were quantified by Griess assay. Results are representative for five independent experiments. *p < 0.05 vs. macrophage control (M2); #*p* < 0.05, &*p* < 0.01. IL interleukin, M macrophage, MSCs mesenchymal stem cells, iNOS inducible nitric oxide synthase, NO_2_ nitrite

To elaborate MSC-triggered effects on M2 polarization, same experiments as described above were performed in the presence of IL-4, which favors M2a polarization. The number of CD86 positive cells was significantly upregulated by primed MSCs when compared to unstimulated MSCs, but no alterations regarding CD206 expression were found (Fig. 3a). In addition, upregulation of intracellular iNOS protein expression in macrophages (Fig. 3a) and increase in NO secretion (Fig. 3b) could be observed in the presence of primed MSCs indicating M0-M2b transition. This assumption was further supported by increased IL-10 secretion found in co-cultures (Fig. 3b). Again, these effects did not depend on physical contact between cells.

### Different regulation of macrophage marker genes by preconditioned MSCs under M1- and M2a-polarizing culture conditions

**Fig. 4:**
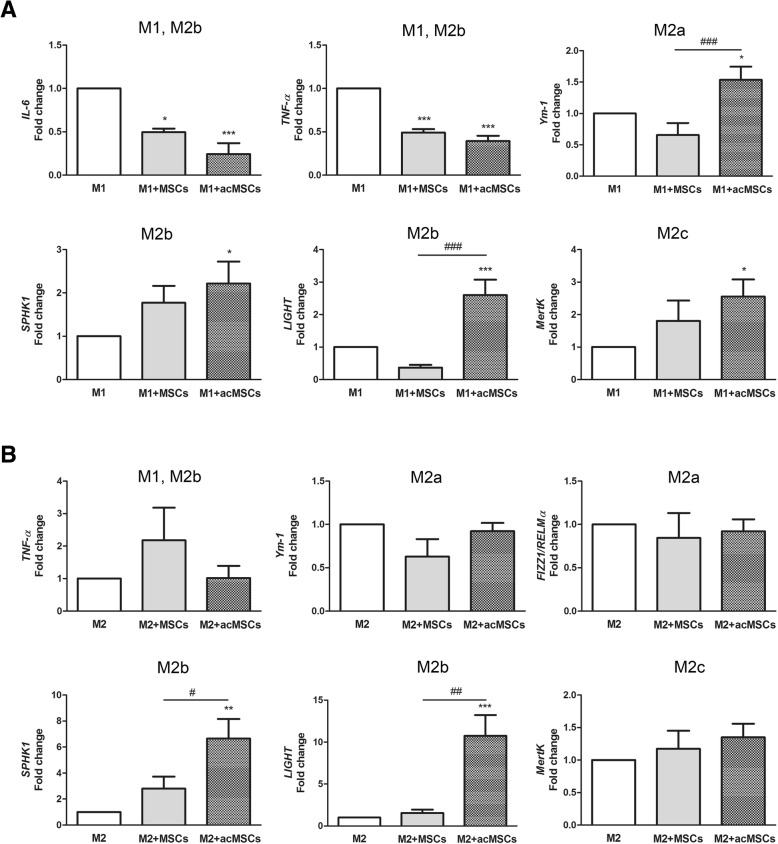
Preconditioned MSCs promote M2 polarization of M1-like cells and M2b polarization of M2a-like cells. MSCs and preconditioned MSCs (acMSCs) were placed in transwells to avoid direct cell interactions and were further co-cultured with differentiated macrophages (M0) under M1- (**a**) and M2- (**b**) polarizing culture conditions for 24 h (ratio 1:2). Marker genes specific for M2a (*Ym-1*), M2b (*SPHK1*, *LIGHT*), and M2c (*MertK*) were analyzed by real-time PCR. In addition, the expression of pro-inflammatory *IL-6* and *TNF-α* were investigated. *n* = 8–10, *p < 0.05, **p < 0.01, ***p < 0.001 vs. control macrophages (M1 and M2). #*p* < 0.05, ##*p* < 0.01, ###*p* < 0.001. FIZZ1/RELMα: found in inflammatory zone 1/resistin-like molecule alpha, IL interleukin, LIGHT lymphotoxin-like, exhibits inducible expression and competes with HSV glycoprotein D for herpes virus entry mediator, a receptor expressed by T lymphocytes, M macrophage, MertK tyrosine-protein kinase MER, MSCs mesenchymal stem cells, acMSCs activated, preconditioned mesenchymal stem cells, SPHK1, sphingosine kinase 1, TNF-α tumor necrosis factor alpha, Ym-1 chitinase-like protein

In order to prove whether preconditioned MSCs indeed promote the polarization toward M2b cells in our experimental setting, gene expression of individual M2 subtype markers was analyzed by real-time PCR. As MSC-derived effects were found to be mediated by soluble mediators, cells were cultured in transwells. According to our assumption, significant suppression of *TNF-α* and *IL-6* gene expression was confirmed under M1-polarizing conditions (Fig. 4a). Additionally, significant upregulation of the M2a marker *Ym-1* [28], the M2b markers *SPHK1* and *LIGHT* [13] and also of *MertK*, which is highly expressed in M2c macrophages [29], has been observed under these conditions. In turn, under M2a-polarizing conditions, MSCs forced specific M2b polarization by upregulating *SPHK1* and *LIGHT* gene expression (Fig. 4b). Notably, without preconditioning, MSCs failed to modulate macrophage polarization although significant inhibition of *IL-6* and *TNF-α* expression could be detected. Only weak, irregular expression of *FIZZ1/RELMα* [30] was detected in the presence IFN-ɣ and LPS (M1 polarization) whereas IL-6 expression was not detected in cultures supplemented with IL-4 (M2a) (not shown).

### Lack of IL-6 receptor in macrophages, but not limited availability of NO and PGE2, prevents M2b polarization in the presence of preconditioned MSCs and IL-4

Immunosuppressive effects of MSCs were previously reported to occur through the secretion of NO, TGF-ß, IL-6, and PGE2, among others. In order to better evaluate the role of these soluble mediators, we used macrophages deficient  in IL-6Rα and cultured them with preconditioned MSCs. In addition, *iNOS* and *COX-2* expression in MSCs was downregulated by siRNA transfection before co-culturing with macrophages under M1- and M2a-polarizing conditions. Since preconditioned MSCs did not secrete higher levels of TGF-ß, the role of TGF-ß was considered as negligible.

**Fig. 5:**
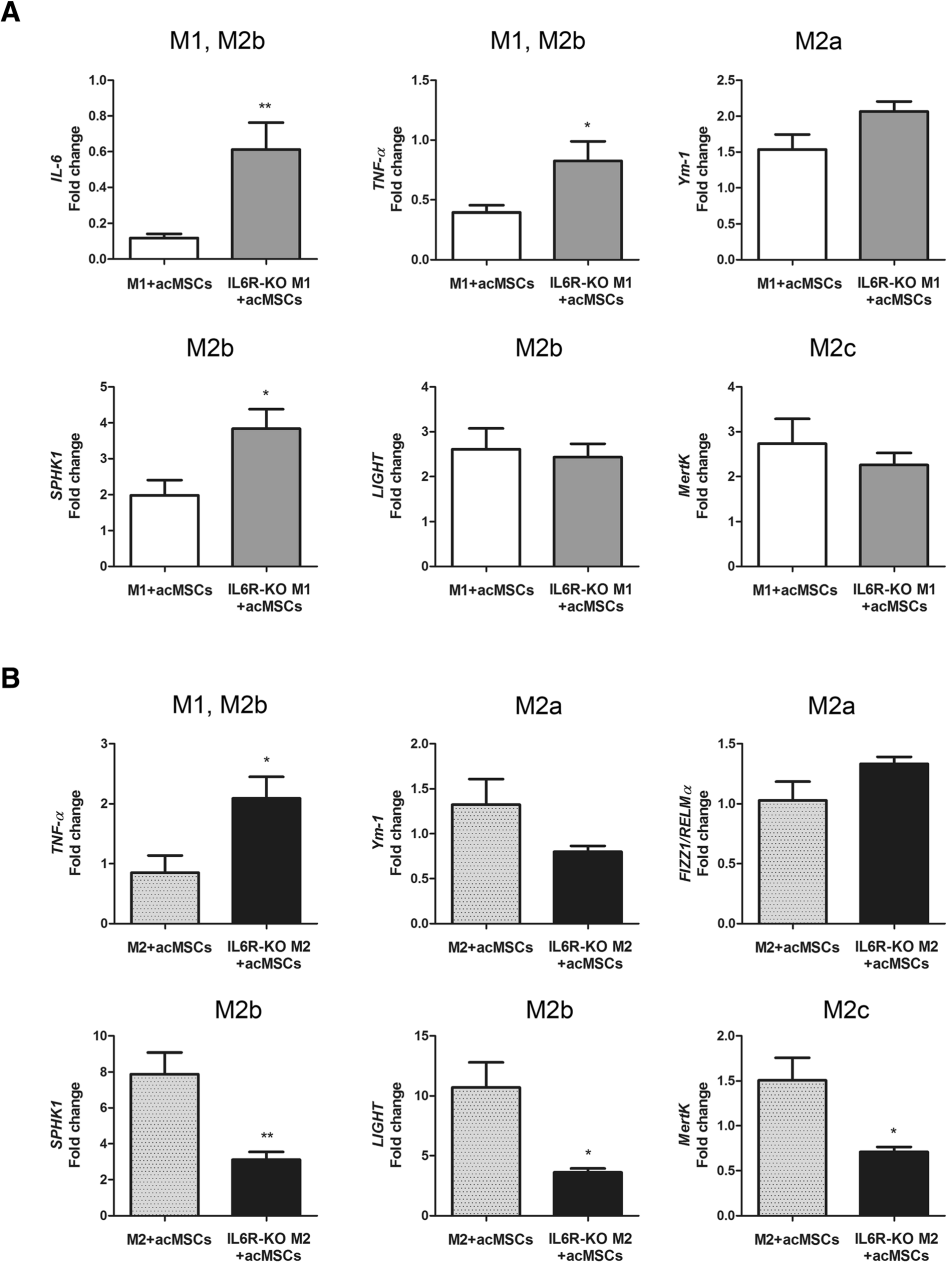
MSC-dependent modulation of macrophage plasticity in IL6Rα-deficient cells. Preconditioned MSCs (acMSCs) were co-cultured with wild type macrophages or macrophages isolated from the bone marrow of IL-6Rα-deficient (IL6R-KO) mice. For differentiation toward an M1-like and M2a-like phenotype, cells were treated with 20 ng/ml IFN-ɣ + 100 ng/ml LPS (**a**) or 20 ng/ml IL-4 (**b**), respectively, for 24 h. Gene expression of *IL-6*, *TNF-α*, M2a markers (*Ym-1*, *FIZZ1/RELMα*), M2b markers (*SPHK1*, *LIGHT*), and the M2c marker *MertK* in macrophages was analyzed by real-time PCR. Results are representative for six to nine independent experiments and are expressed as fold change vs. expression found in macrophages. *p < 0.05, **p < 0.01 vs. gene expression found in wild type macrophages (M1 or M2) co-cultured with preconditioned MSCs (acMSCs). FIZZ1/RELMα: found in inflammatory zone 1/resistin-like molecule alpha, IL interleukin, IL-6Rα interleukin 6 receptor alpha, KO knockout, LIGHT lymphotoxin-like, exhibits inducible expression and competes with HSV glycoprotein D for herpes virus entry mediator, a receptor expressed by T lymphocytes, M macrophage, MertK tyrosine-protein kinase MER, MSCs mesenchymal stem cells, acMSCs activated, preconditioned mesenchymal stem cells, SPHK1, sphingosine kinase 1, TNF-α tumor necrosis factor alpha, Ym-1 chitinase-like protein

Absence of IL-6Rα on macrophages [14] has been confirmed by flow cytometry (Additional file 3: Figure S3a). Additionally, macrophages isolated from IL-6Rα-deficient C57Bl6 mice displayed widely same characteristics after polarization as wild type macrophages. However, *SPHK1* gene expression in M1-polarized cells was significantly increased (Additional file 3: Figure S3b). Cells deficient  in IL-6Rα further showed strong suppression of *FIZZ1/RELMα* expression after stimulation with IL-4 when compared to wild type macrophages whereby *Ym1*, which represents an additional M2a signature gene, did not change (Additional file 3: Figure S3c). Data presented in Fig. 5 demonstrate that secreted IL-6 profoundly suppresses *IL-6* and *TNF-α* expression in macrophages under M1-polarizing conditions, as the expression of both genes was significantly upregulated in macrophages deficient  in IL-6Rα compared to wild type cells (Fig. 5a). Having found that IL-6R-deficient M1-polarized cells express higher levels of *SPHK1* mRNA, this gene does not seem to be regulated by co-cultured MSCs. We also found that lack of IL-6Rα under M2a-polarizing conditions strongly affects the expression of *SPHK1*, *LIGHT*, and *MertK* indicating that MSC-derived IL-6 represents an important mediator of M2b and probably M2c polarization (Fig. 5b).

**Fig. 6:**
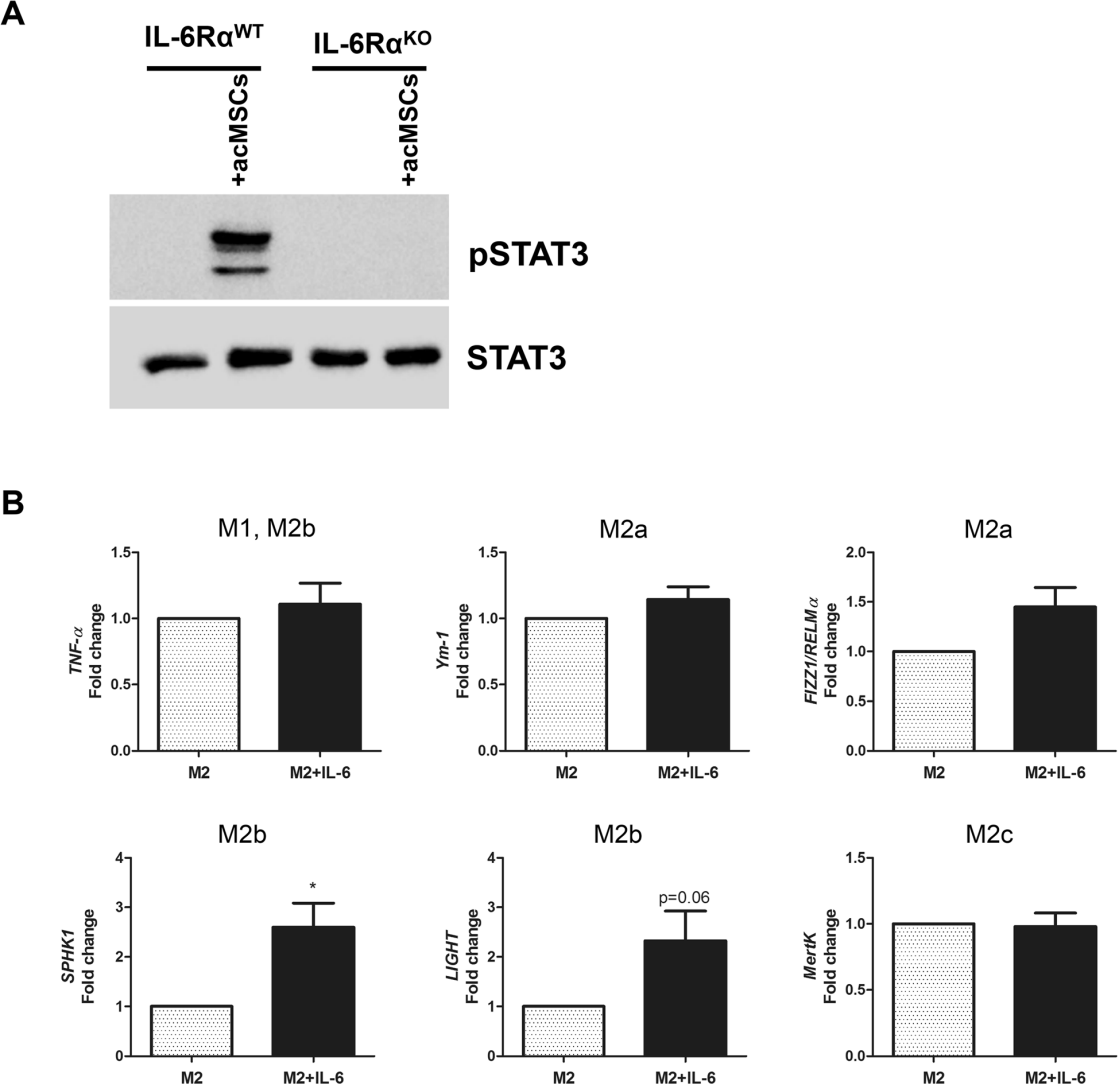
Role of IL-6 for macrophage polarization toward M2b phenotype. **a** Preconditioned MSCs (acMSCs) were co-cultured with wild type macrophages or macrophages isolated from IL-6Rα-deficient (IL6R-KO) mice in the presence of IL-4 (20 ng/ml). After 18 h, the expression of pSTAT3 (Tyr705) was verified by Western blot. STAT3 served as loading control. One representative Western blot of three independent experiments is depicted. **b** Wild type macrophages were treated with 20 ng/ml IL-4 and 25 ng/ml IL-6 for 24 h. Then, the expression of macrophage-specific markers and *TNF-α* was analyzed by real-time PCR. *n* = 6, *p < 0.05. FIZZ1/RELMα, found in inflammatory zone 1/resistin-like molecule alpha, IL interleukin, IL-6Rα interleukin 6 receptor alpha, KO knockout, LIGHT lymphotoxin-like, exhibits inducible expression and competes with HSV glycoprotein D for herpes virus entry mediator, a receptor expressed by T lymphocytes, M macrophage, MertK tyrosine-protein kinase MER, MSCs mesenchymal stem cells, acMSCs activated, preconditioned mesenchymal stem cells, SPHK1 sphingosine kinase 1, STAT3 Signal transducer and activator of transcription 3, pSTAT3 phosphorylated STAT3, TNF-α tumor necrosis factor alpha, WT wild type, Ym-1 chitinase-like protein

To validate the role of IL-6 signaling for MSC-mediated M2b polarization, we performed Western blot analysis to detect phosphorylated STAT3, which is implicated in the IL-6 transduction pathway. Indeed, cells deficient  in IL-6Rα failed to upregulate pSTAT3 upon incubation with IL-6-secreting MSCs, confirming an impaired IL-6 signaling pathway in these cells (Fig. 6a). Of note, recombinant IL-6 widely mimicked the effects mediated by preconditioned MSCs. As displayed in Fig. 6b, in the presence of IL-4 and IL-6, macrophages significantly upregulated *SPHK1* expression and to a lesser, not significant, extent *LIGHT* expression. Thus, as M2b polarization was strongest in the presence of preconditioned MSCs, it is likely that M2b polarization does not solely depend on IL-6 expression.

**Fig. 7:**
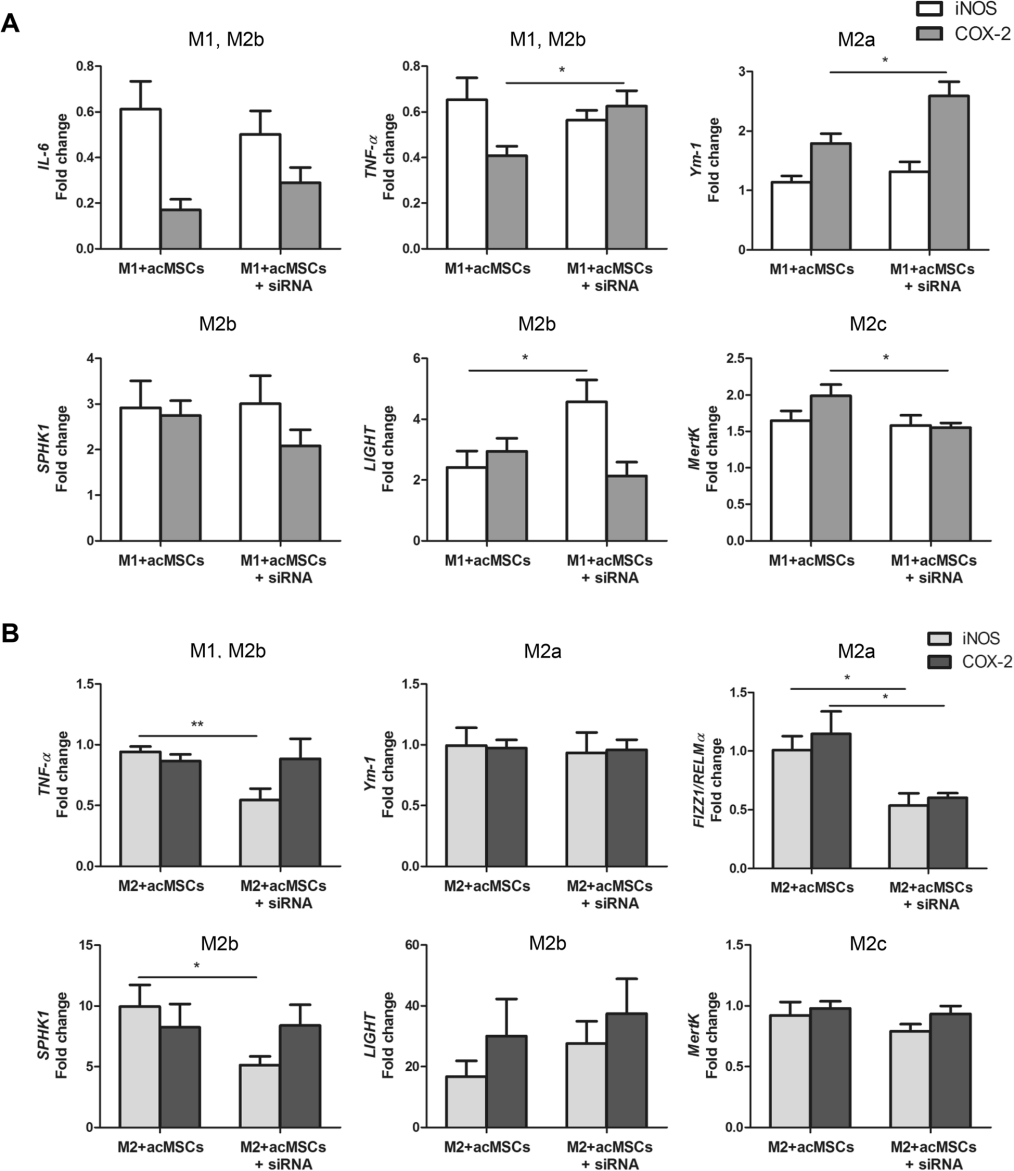
NO and PGE2 are only partially involved in MSC-mediated macrophage polarization. MSCs were transfected with *iNOS-* or *COX-2-*specific siRNAs to reduce NO and PGE2 secretion and further stimulated with IFN-ɣ and IL-1ß. Transfected, activated MSCs (acMSCs+siRNA) were co-cultured with M0 macrophages under M1- (20 ng/ml IFN-gamma + 100 ng/ml LPS, **a**) and M2a- (20 ng/ml IL-4, **b**) polarizing culture conditions for 24 h. Preconditioned MSCs (acMSCs) served as controls. Gene expression of *IL-6*, *TNF-α*, M2a markers (*Ym-1*, *FIZZ1/RELMα*), M2b markers (*SPHK1*, *LIGHT*), and the M2c marker *MertK* in macrophages was analyzed by real-time PCR. Results are representative for six to eight independent experiments and are expressed as fold change vs. expression found in macrophages. *p < 0.05, **p < 0.01. FIZZ1/RELMα, found in inflammatory zone 1/resistin-like molecule alpha, IL interleukin, LIGHT lymphotoxin-like, exhibits inducible expression and competes with HSV glycoprotein D for herpes virus entry mediator, a receptor expressed by T lymphocytes, M macrophage, MertK tyrosine-protein kinase MER, MSCs mesenchymal stem cells, acMSCs activated, preconditioned mesenchymal stem cells, SPHK1, sphingosine kinase 1, TNF-α tumor necrosis factor alpha, Ym-1 chitinase-like protein

To further elaborate the role of MSC-derived NO and PGE2 for macrophage polarization, MSCs were transfected with *iNOS* as well as *COX-2* specific siRNA before preconditioning. Knockdown efficiency of both genes in transfected, preconditioned MSCs was verified by real-time PCR (Additional file 4: Figure S4a). *COX-2* knockdown did not influence NO nor IL-6 secretion by MSCs. Additionally, PGE2 as well as IL-6 secretion were not altered after transfection of MSCs with *iNOS* siRNA. Consistently, significant reduction of NO and PGE2 could be detected in *iNOS* or *COX-2* siRNA-transfected MSCs, respectively (Additional file 4: Figure S4b). Impaired NO availability under M1-polarizing conditions resulted in increased *LIGHT* expression, indicating that this gene is usually downregulated by NO. Similarly, PGE2 was found to downregulate *TNF-α* and *Ym-1* (M2a marker) under same conditions. However, MSC-derived PGE2 seemed to promote M2c polarization, as *MertK* was significantly reduced in macrophages co-cultured with *COX-2* transfected MSCs (Fig. 7a). NO was further found to be an inducer of *TNF-α* expression in the presence of IL-4 and to further upregulate *FIZZ1/RELMα* (M2a) (Fig. 7b). The last one was also identified to be reduced in the absence of PGE2. Thus, although both NO and PGE2 drive the expression of the M2a-specific gene *FIZZ/RELMα*, they play a subordinate role in MSC-mediated M2b polarization.

### M2b macrophages suppress IFN-ɣ production by CD4^+^ T lymphocytes more efficiently than M2a macrophages

**Fig. 8:**
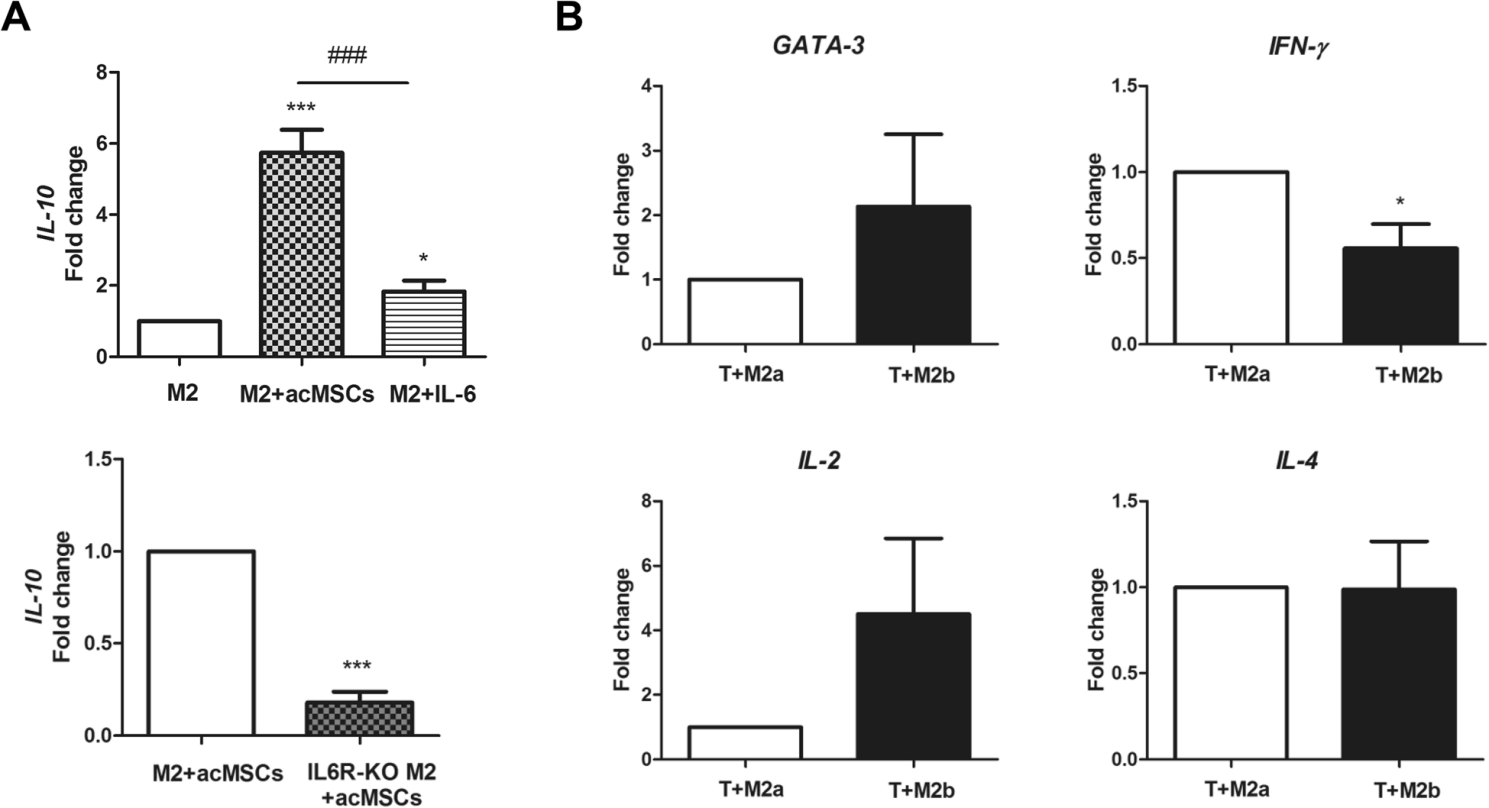
M2b-polarized macrophages produce high levels of IL-10 and impair *IFN-ɣ* expression in CD4^+^ naïve T lymphocytes. **a** *IL-10* gene expression was proven in macrophages isolated from wild type macrophages (M2) after co-culture with preconditioned MSCs and IL-4 or treatment with recombinant IL-4 (20 mg/ml) + IL-6 (25 ng/ml), respectively. Expression of *IL-10* in cells from IL-6Rα-deficient mice (IL6R-KO M2) after co-culture with preconditioned MSCs + IL-4 was additionally analyzed. Fold change in gene expression vs. control cells is depicted. *n* = 6, *p < 0.05; ***p < 0.001. **b** Wild type macrophages pre-cultured with or without preconditioned MSCs and IL-4 for 24 h were further co-cultured with CD4^+^ naïve T lymphocytes allowing direct cell-cell interactions for further 24 h. Gene expression of Th1 markers *GATA-3* and *IFN-ɣ* as well as *IL-2* and *IL-4* (Th2) was analyzed by real-time PCR. *n* = 5, *p < 0.05. IL interleukin, IFN-ɣ interferon gamma, M macrophage, T lymphocyte

To further confirm that MSCs trigger M2b polarization, we investigated the expression of IL-10 in cells previously co-cultured with MSCs. In this regard, it is already known that M2b macrophages produce high levels of IL-10 when compared to other macrophage subtypes [31]. *IL-10* gene expression did not differ between wild type and IL-6Rα-deficient M2-polarized macrophages (not shown). As shown in Fig. 8a, macrophages cultured in the presence of IL-4 and preconditioned MSCs expressed higher levels of *IL-10* compared to M2a-polarized control macrophages. Recombinant IL-6 also increased the expression of *IL-10* in macrophages; however, this increase was significantly reduced when compared to MSC-mediated *IL-10* upregulation. Correspondingly, macrophages from IL-6Rα-deficient mice showed strong reduction in *IL-10* expression after co-culturing with preconditioned MSCs (versus wild type macrophages), arguing against an M2b phenotype.

We next questioned if macrophages cultured in the presence of IL-4 and preconditioned MSCs might influence Th1/Th2 differentiation of naïve CD4^+^ T lymphocytes. To this end, we cultured M2a-polarized and macrophages pre-cultured with preconditioned MSCs (M2b phenotype) with CD4^+^ T cells (Additional file 5) for 24 h in the presence of CD3 and CD28 antibodies. Then, gene expression of *GATA-3*, *IL-4*, *IFN-ɣ*, and *IL-2* was analyzed by real-time PCR to discriminate between Th1 and Th2 lymphocytes. As shown in Fig. 8b, T cells co-cultured with M2b macrophages showed significantly reduced *IFN-ɣ* expression compared to cells cultured in the presence of M2a macrophages. Further on, higher not significant *GATA-3* and *IL-2* expression could be detected, indicating that M2b cells significantly suppress pro-inflammatory pathways presumably favoring Th2 differentiation.

## Discussion

In the recent past, the promising immunomodulatory effects of MSCs and their therapeutic potential have become evident. However, beside first encouraging results in clinical studies, most MSC-based clinical trials occur in an early phase (phase I-II), demonstrating that the therapeutic effectiveness of MSCs needs to be investigated. Among all MSC-based studies, there is a substantial proportion dealing with the treatment of cardiovascular diseases. Because ischemic heart diseases and heart failure remain major causes of morbidity and mortality, much effort is expanded to repair cardiac tissue with stem cell transplantation. The therapeutic properties of MSCs are largely related to their anti-inflammatory and immunomodulatory abilities which have been confirmed by in vivo and in vitro studies [8, 10, 32]. However, low survival rate in vivo and undefined mechanisms underlying the beneficial properties of MSCs represent crucial reasons accounting for the impeded cardiac repair effect of MSCs.

Impaired pro-inflammatory response of macrophages and changes in the M1/M2 balance in the presence of MSCs has already been evidenced. Macrophage polarization is critical for the resolution of inflammation and tissue remodeling [33]. During the first days after myocardial infarction, neutrophils and M1 macrophages dominate in the infarcted area, whereas M2 transition during the late stage triggers Th2 responses and resolution of inflammation. Prolonged presence of M1 macrophages extends the pro-inflammatory environment and causes expansion of the infarcted area in the myocardium post-acute myocardial infarction (AMI) [34]. In turn, macrophage polarization toward the M2 phenotype has been shown to promote the resolution of inflammation and improve infarct healing post-AMI [35]. Therefore, elucidation of MSC-triggered mechanisms and improvement of their anti-inflammatory capacities represent an attractive strategy for MSC-based therapies.

In this study, we found that preconditioning of murine MSCs with IFN-ɣ and IL-1ß highly improves their potential to promote immunomodulation. In fact, preconditioning of MSCs resulted in significantly elevated NO, IL-6, and PGE2 production, which have been already described to represent prominent immunosuppressive molecules [36, 37], although their mode of action remains obscure. Preconditioned MSCs markedly reduced CD86, iNOS protein expression, and TNF-α secretion by macrophages when cultured in the presence of pro-inflammatory IFN-ɣ and LPS (M1 polarization). On the other hand, under M2a-polarizing culture conditions (IL-4), upregulation of CD86, iNOS, and increased NO and IL-10 secretion could be found. According to previously published results, these effects largely depended on paracrine factors and not on direct cell-to-cell contact [38]. Our data strongly suggest that immunoregulation by MSCs under pro-inflammatory conditions, especially after preconditioning with IFN-ɣ and IL-1ß, is based on a shift from the inflammatory M1 phenotype toward the anti-inflammatory M2 phenotype. In this regard, it was already reported that different M2 macrophage subtypes, namely M2a, M2b, and M2c, exhibiting different functions exist. Whereas M2a/c macrophages were found to be beneficial in early inflammatory stages, they have been found to impair tissue remodeling [39]. M2b cells have been proposed to be immunoregulatory diminishing immune responses with minor damage to local tissue [40]. Having found that M2-polarized macrophages upregulated CD86 expression and NO and IL-10 production, we propose that preconditioned MSCs are effective in M2b polarization in the presence of anti-inflammatory IL-4. Indeed, by analyzing gene expression of selected macrophage markers after co-culture with preconditioned MSCs, we found specific significant upregulation of the M2b markers *SPHK1* and *LIGHT* in macrophages in the presence of the M2a inducer IL-4. In turn, all M2 markers were upregulated under M1-polarizing co-culture conditions. Notably, although unprimed MSCs were able to reduce the expression of pro-inflammatory *IL-6* and *TNF-α* in macrophages, these cells failed to modulate M2 macrophage marker expression, thus being in line with recently reported data [41].

We also showed that macrophages co-cultured with preconditioned MSCs in the presence of IL-4 displayed highest IL-10 production, which could be attributed to the M2b phenotype initiated by preconditioned MSCs. In this regard, when compared with other macrophage subtypes, M2b cells are known to represent the main cellular source for IL-10, playing an important role in the resolution of inflammation [31]. Direct co-culture of IL-10-producing M2b macrophages resulted in significant downregulation of *IFN-ɣ* expression in CD4^+^ T lymphocytes and also slight increase in expression of the Th2 marker *GATA-3* [42], indicating polarization toward a Th2 phenotype and suppression of Th1 responses.

Previous studies argue for a prominent role of PGE2 as a key immunosuppressive mediator derived from MSCs [36, 41, 43]. Additionally, both PGE2 and NO production are positively regulated by IL-6 and their secretion is strongly reduced in the absence of IL-6. In light of these considerations, we further elucidated the role of IL-6, NO, and PGE2 for MSC-mediated M2 or M2b polarization, respectively. Using IL-6Rα-deficient macrophages, we demonstrate here that IL-6 is the main MSC-derived mediator of *IL-6* and *TNF-α* suppression under M1-polarizing conditions. Hence, MSCs failed to upregulate almost all M2 marker genes in cells cultured in the presence of M1 inducers lacking IL-6 signaling. Most importantly, IL-6Rα-deficient macrophages showed upregulated *TNF-α* expression and significantly reduced *SPHK1*, *LIGHT*, and *MertK* expression after co-culturing with preconditioned MSCs, suggesting that IL-6 represents an essential regulator of M2b and probably M2c polarization. Indeed, after co-culture with preconditioned MSCs, macrophages deficient in IL-6Rα expressed strongly suppressed levels of IL-10 when compared to wild type cells, most likely due to impaired IL-6 signaling as evidenced by suppressed STAT3 activation. Although there is already evidence for the role of IL-6 for the polarization of alternatively activated macrophages [44, 45], in our knowledge, this study is the first showing that MSC-derived IL-6 triggers polarization toward the M2b phenotype. Thus, our results strongly underline the recently postulated anti-inflammatory role of IL-6 [44, 46]. Nevertheless, our data with recombinant IL-6 implicate that additional MSC-derived soluble factors might also be involved in M2b polarization. In contrast to previously published work [31, 43], PGE2 was not found to influence macrophage polarization or to favor M2b polarization. *COX-2* knockdown indeed strongly reduced PGE2 secretion, without affecting NO or IL-6 secretion by preconditioned MSCs, but this impaired PGE2 production did not diminish the expression of M2 marker genes, except for *FIZZ/RELMα*. These discrepancies between results might be based on different experimental setups used. In this regard, in vitro polarized macrophage subsets [43] and combination of PGE2 with LPS [31] have been proposed for the induction of regulatory macrophages. Further on, the capacity of MSCs to regulate diverse macrophage features was demonstrated to differ between various strains of mice [36]. In addition, our data confirmed that MSC-mediated generation of regulatory macrophages is largely independent on NO secretion [36].

Altogether, our results presented here strongly suggest that MSCs, beside their potency to inhibit T cell proliferation [47] and to promote generation of Treg [48], significantly influence the plasticity of macrophages promoting a regulatory phenotype. Unpublished data of our group revealed pro-proliferative effects of preconditioned MSCs on macrophages, although the impact if IL-6 has not been further evaluated. Also, a possible link between M2b polarization and increased proliferative activity should be considered in further studies. The modulation of macrophage plasticity represents an important therapeutic strategy [49]. In the early stages of inflammation, MSCs might limit M1 responses and trigger M2 polarization, whereas during the late anti-inflammatory response, they might promote the generation of a regulatory M2b phenotype via IL-6 secretion. In view of these results, we propose that therapeutic use of preconditioned MSCs might be beneficial during different stages of inflammation in cardiovascular diseases. Indeed, in a very recent study, transplantation of M2b macrophages significantly ameliorated myocardial ischemia/reperfusion injury in mice [40]. Further in vivo studies based on preconditioned MSCs should be conducted in the future to enhance immunosuppressive capacity of MSCs and M2b polarization and to validate the results of this study.

## Conclusions

Preconditioning of MSCs with IL-1ß and IFN-ɣ strongly improves the capacity of MSCs to modulate macrophage plasticity, mainly by IL-6 secretion. MSC-dependent polarization toward the M2 phenotype in a pro-inflammatory microenvironment and toward the regulatory M2b phenotype in the presence of anti-inflammatory IL-4 should be considered for future therapeutic approaches to treat cardiovascular diseases.

## Additional files

Additional file 1: **Figure S1.** Characterization of bone-marrow derived MSCs. (a) As assessed by flow cytometry, MSCs were positive for the well-established MSCs markers CD29, CD44, CD49e and Sca-1 and negative for CD11b and CD45. (b) Adipogenic, chondrogenic and osteogenic differentiation of MSCs. Scale bar, 100 μm. (TIF 9562 kb)

Additional file 2: **Figure S2.** Characterization of M0, M1- and M2a-like macrophages. (a) CD11b and F4/80 expression on bone marrow-derived differentiated M0 macrophages. M0 macrophages were further polarized in vitro to M1-like and M2a-like cells by treatment with 20 ng/ml IFN-ɣ and 100 ng/ml LPS (M1) or 20 ng/ml IL-4 (M2), respectively, for 24 h. (b) Gene expression of *iNOS* and *Arg I* was determined by real-time PCR. *n* = 3. (c) TNF-α and IL-6 levels in culture supernatants were quantified by Elisa. *n* = 5, *p < 0.05, **p < 0.001. (TIF 1040 kb)

Additional file 3: **Figure S3.** Characterization of IL-6Rα-deficient macrophages. (a) IL-6Rα expression on bone marrow-derived M0 macrophages from WT and IL-6Rα-deficient mice. *n* = 3, **p < 0.01. (b) IL-6Rα-deficient and wild  type macrophages were differentiated into M1- and M2a-like cells as described in Methods. Gene expression of *IL-6*, *TNF-α* and the subtype-specific markers *Ym-1*, *FIZZ1/RELMα* (M2a markers), *SPHK1*, *LIGHT* (M2b markers) and *MertK* (M2c marker) was determined by real-time PCR. *n* = 4–6, *p < 0.05, ***p < 0.001. (TIF 2221 kb)

Additional file 4: **Figure S4.** siRNA knockdown of *iNOS* and *COX-2* expression in preconditioned MSCs. MSCs were transfected with siRNA specific for *iNOS*, *COX-2* or control siRNA (each 5.5 nM), respectively. After 24 h, cells were preconditioned with IFN-ɣ and IL-1ß. (a) Transfection efficiency was proven by real-time PCR. *n* = 5, **p < 0.01 vs. control siRNA (Ctrl). ##*p* < 0.01, ###*p* < 0.001. (b) Levels of NO, IL-6 and PGE2 secreted by transfected MSCs were determined by Griess assay and Elisa. *n* = 5–6, *p < 0.05, ***p < 0.001, n.s. not significant (TIF 1510 kb)

Additional file 5: **Figure S5.** Flow cytometric analysis of CD4 expression on isolated T lymphocytes. (TIF 429 kb)

## Abbreviations

AMI: Acute myocardial infarction
EGF: Epidermal growth factor
FGF: Fibroblast growth factor
FIZZ1/RELMα: Found in inflammatory zone 1/resistin-like molecule
HGF: Hepatocyte growth factor
IFN-ɣ: Interferon gamma
IGF: Insulin-like growth factor
IL: Interleukin
IL-6Rα: Interleukin 6 receptor alpha
iNOS: Inducible nitric oxide synthase
LIGHT: Lymphotoxin-like, exhibits inducible expression and competes with HSV glycoprotein D for herpes virus entry mediator, a receptor expressed by T lymphocytes
LPS: Lipopolysaccharides
MertK: Tyrosine-protein kinase MER
MSCs: Mesenchymal stem cells
PDGF: Platelet-derived growth factor
PGE2: Prostaglandin E2
SDF: Stromal cell-derived factor
SPHK1: Sphingosine kinase 1
STAT3: Signal transducer and activator of transcription 3
pSTAT3: Phosphorylated STAT3
TGF-ß: Transforming growth factor beta
TNF-α: Tumor necrosis factor alpha
VEGF: Vascular endothelial growth factor
Ym-1: Chitinase-like protein
