# Supplementary figures and images for: Preconditioning of bone marrow-derived mesenchymal stem cells highly strengthens their potential to promote IL-6-dependent M2b polarization
Source: Stem Cell Res Ther. 2018 Oct 25;9:286. doi: 10.1186/s13287-018-1039-2 (PMC6202843; doi:10.1186/s13287-018-1039-2)

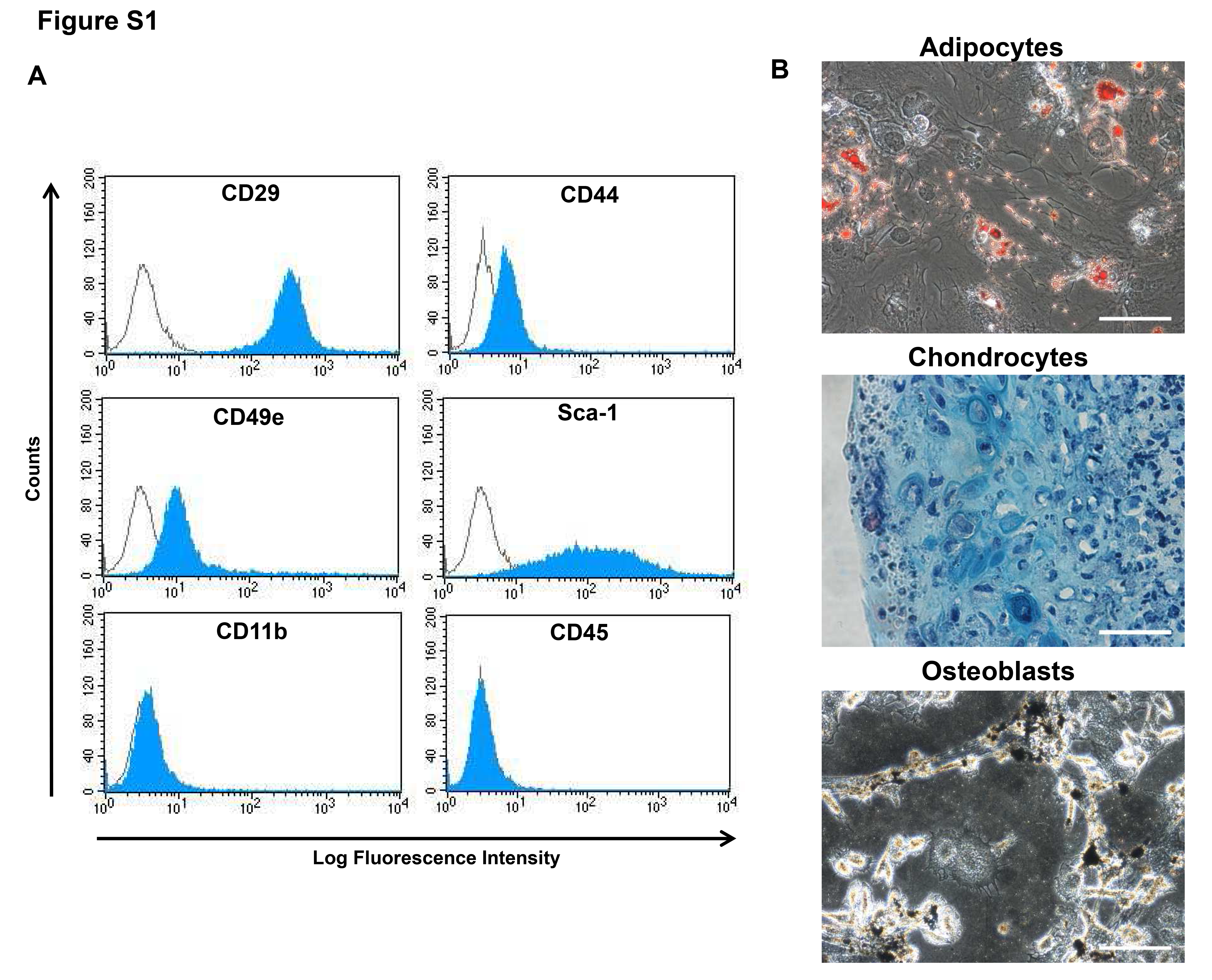

Supplement: Supplementary file 1 — Figure S1. Characterization of bone-marrow derived MSCs. (a) As assessed by flow cytometry, MSCs were positive for the well-established MSCs markers CD29, CD44, CD49e and Sca-1 and negative for CD11b and CD45. (b) Adipogenic, chondrogenic and osteogenic differentiation of MSCs. Scale bar, 100 μm. (TIF 9562 kb) [file 13287_2018_1039_MOESM1_ESM.tif]

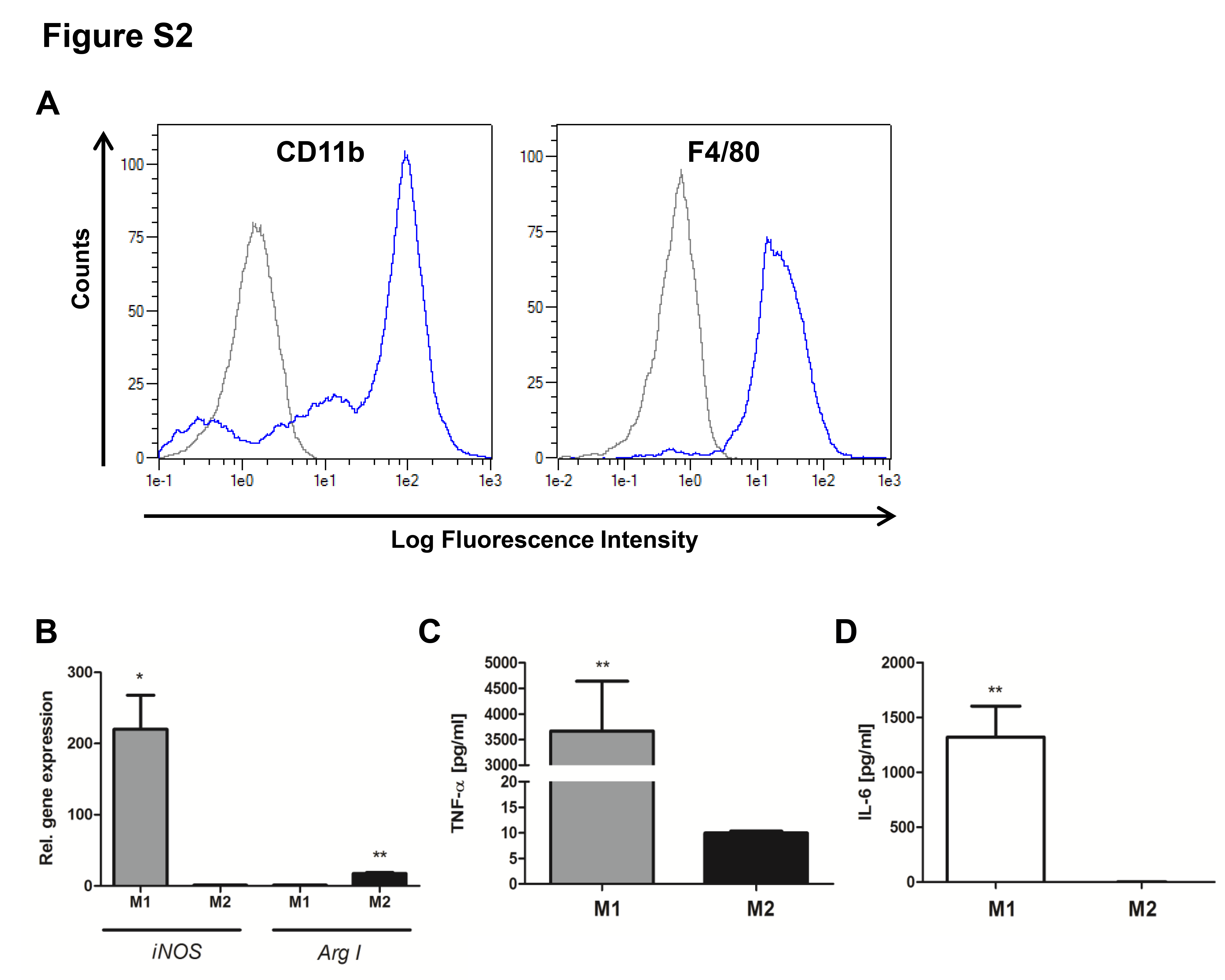

Supplement: Supplementary file 2 — Figure S2. Characterization of M0, M1- and M2a-like macrophages. (a) CD11b and F4/80 expression on bone marrow-derived differentiated M0 macrophages. M0 macrophages were further polarized in vitro to M1-like and M2a-like cells by treatment with 20 ng/ml IFN-ɣ and 100 ng/ml LPS (M1) or 20 ng/ml IL-4 (M2), respectively, for 24 h. (b) Gene expression of iNOS and Arg I was determined by real-time PCR. n = 3. (c) TNF-α and IL-6 levels in culture supernatants were quantified by Elisa. n = 5, *p < 0.05, **p < 0.001. (TIF 1040 kb) [file 13287_2018_1039_MOESM2_ESM.tif]

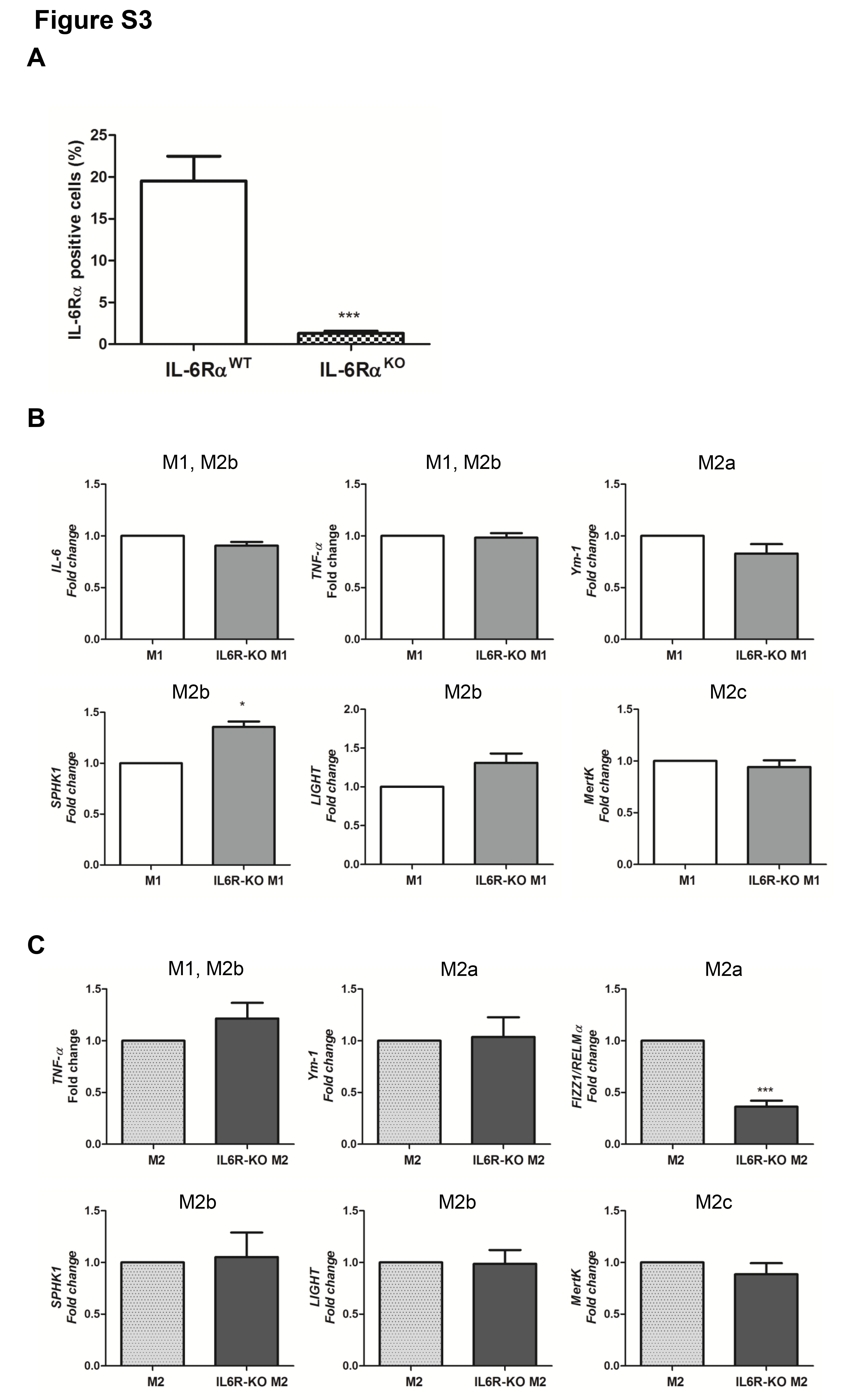

Supplement: Supplementary file 3 — Figure S3. Characterization of IL-6Rα-deficient macrophages. (a) IL-6Rα expression on bone marrow-derived M0 macrophages from WT and IL-6Rα-deficient mice. n = 3, **p < 0.01. (b) IL-6Rα-deficient and wild type macrophages were differentiated into M1- and M2a-like cells as described in Methods. Gene expression of IL-6, TNF-α and the subtype-specific markers Ym-1, FIZZ1/RELMα (M2a markers), SPHK1, LIGHT (M2b markers) and MertK (M2c marker) was determined by real-time PCR. n = 4–6, *p < 0.05, ***p < 0.001. (TIF 2221 kb) [file 13287_2018_1039_MOESM3_ESM.tif]

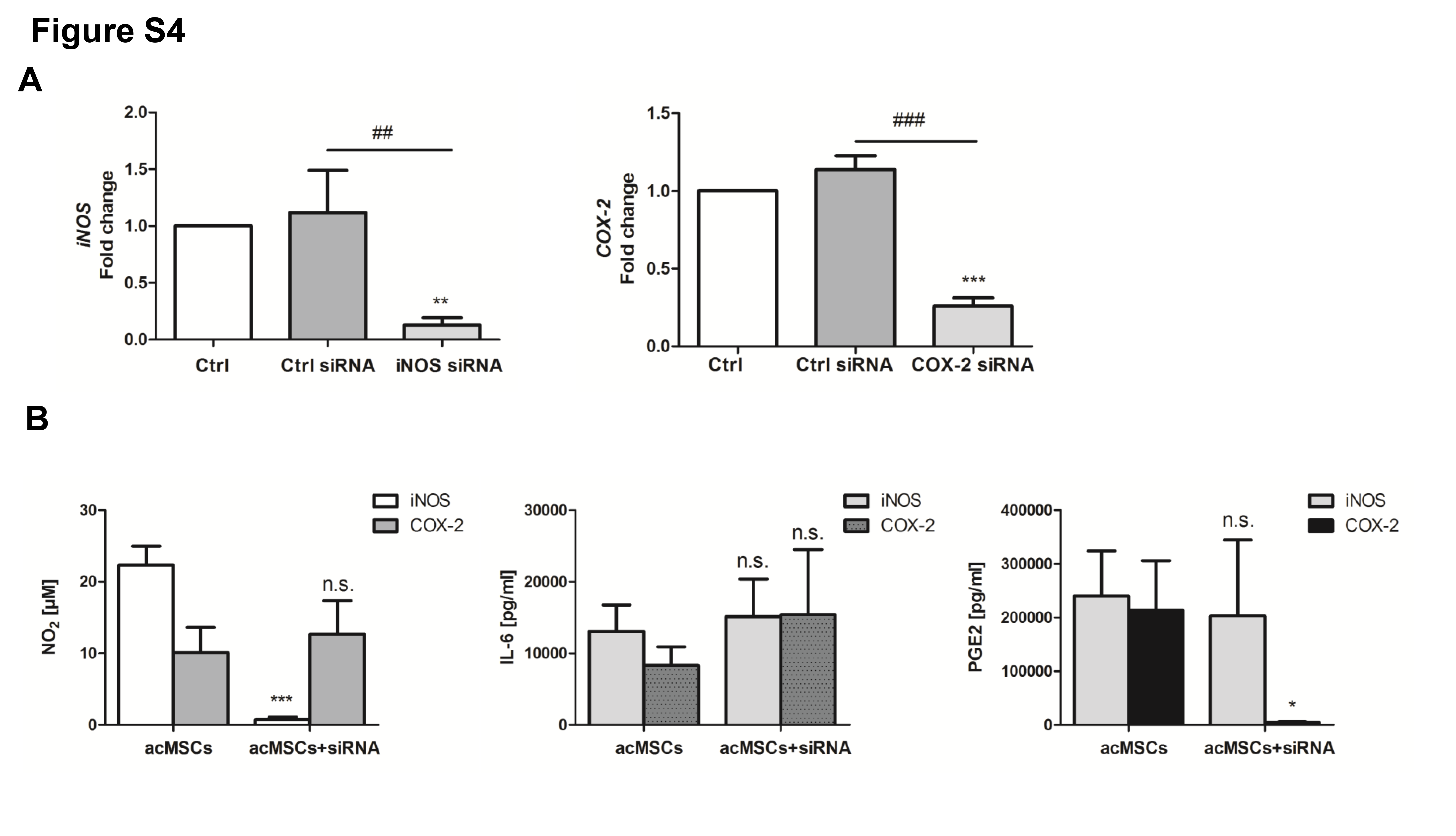

Supplement: Supplementary file 4 — Figure S4. siRNA knockdown of iNOS and COX-2 expression in preconditioned MSCs. MSCs were transfected with siRNA specific for iNOS, COX-2 or control siRNA (each 5.5 nM), respectively. After 24 h, cells were preconditioned with IFN-ɣ and IL-1ß. (a) Transfection efficiency was proven by real-time PCR. n = 5, **p < 0.01 vs. control siRNA (Ctrl). ##p < 0.01, ###p < 0.001. (b) Levels of NO, IL-6 and PGE2 secreted by transfected MSCs were determined by Griess assay and Elisa. n = 5–6, *p < 0.05, ***p < 0.001, n.s. not significant (TIF 1510 kb) [file 13287_2018_1039_MOESM4_ESM.tif]

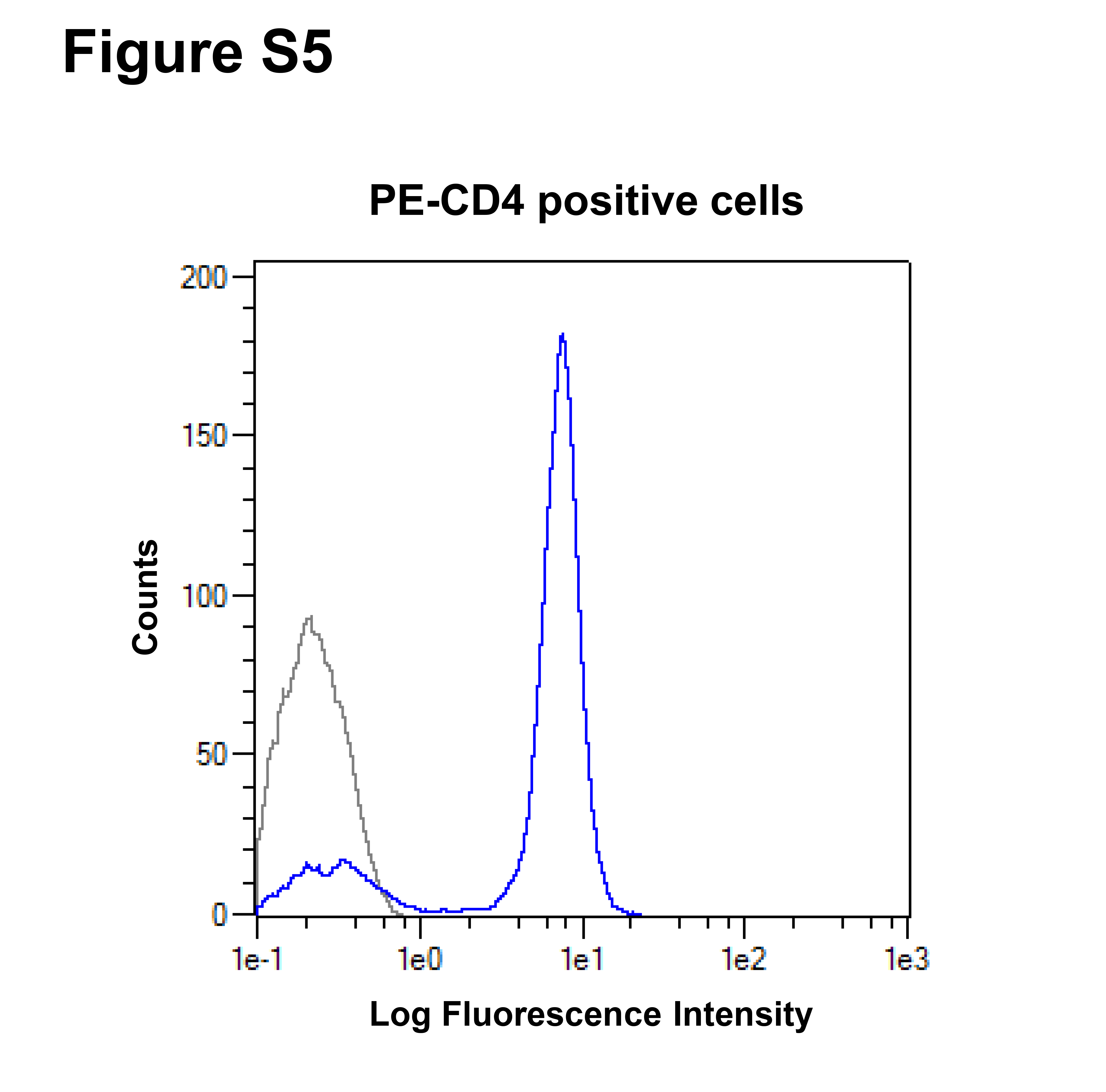

Supplement: Supplementary file 5 — Figure S5. Flow cytometric analysis of CD4 expression on isolated T lymphocytes. (TIF 429 kb) [file 13287_2018_1039_MOESM5_ESM.tif]
